# Supplementary material for: The Lebanese Red Algae Jania rubens: Promising Biomolecules against Colon Cancer Cells
Source: Molecules. 2022 Oct 5;27(19):6617. doi: 10.3390/molecules27196617 (PMC9571134; doi:10.3390/molecules27196617)
Supplement: Supplementary file 1 [file molecules-27-06617-s001.zip › molecules-1926491-supplementary.pdf]

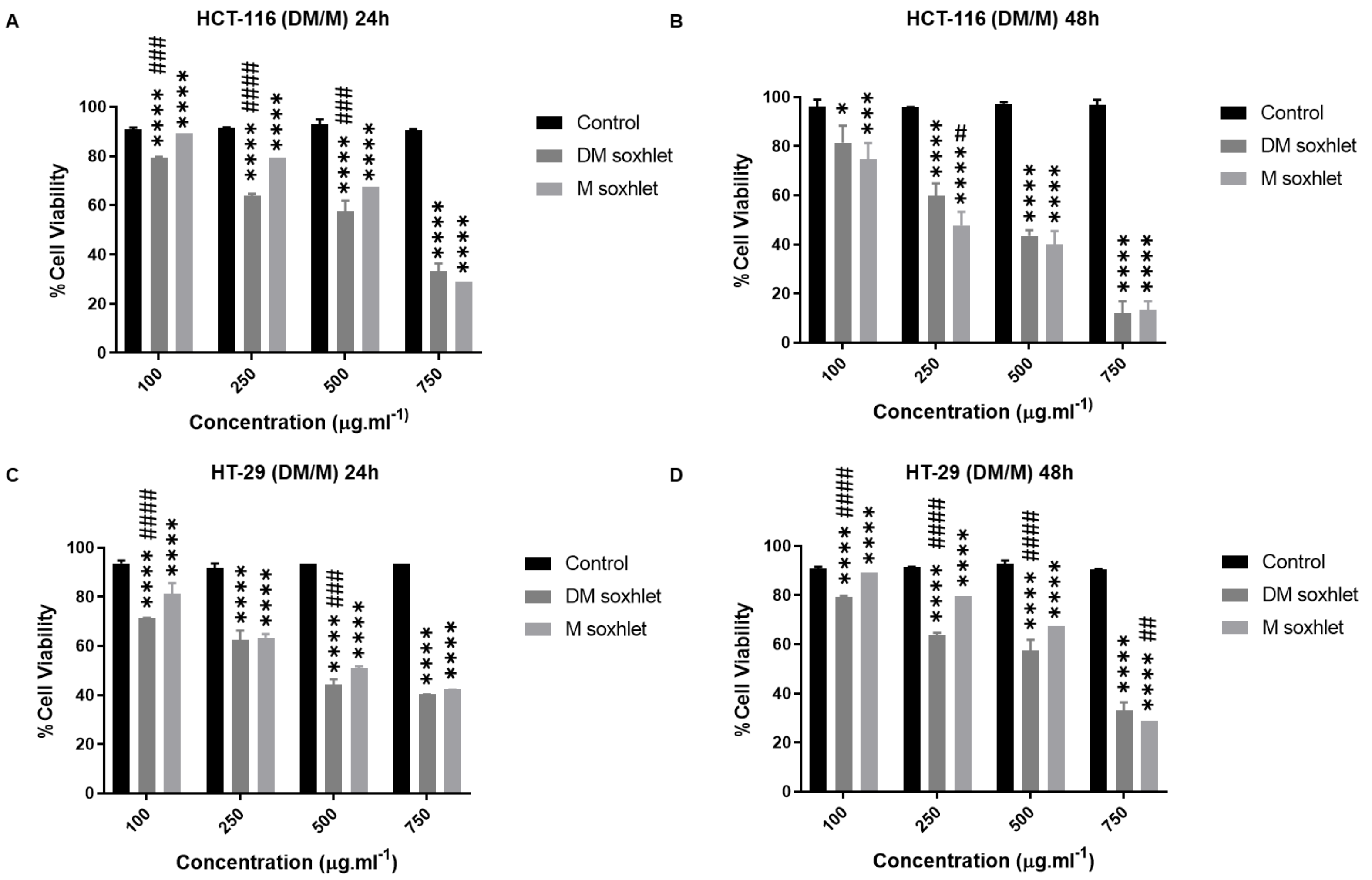

Figure S1. Cytotoxic effect of *J. Rubens* DM and M crude extracts (100-250-500-750 $\mu g.ml^{-1}$) on colon cancer cells using Trypan blue. (**A, B**) Effect on HCT-116 cells at 24h and 48h. (**C, D**) Effect on HT-29 cells at 24h and 48h. Results are present as the mean ± SD (n≥3). *P<0.05 ***P<0.001 ****P<0.0001 vs control group. $^{\#}$P<0.05 $^{\#\#}$P<0.01 $^{\#\#\#}$P<0.001 $^{\#\#\#\#}$P<0.0001 vs DM, Dichloromethane/ methanol; M, methanol.
